# Operative Management of T1b Gallbladder Carcinoma with Concurrent Hepatic Anastomosing Hemangioma

**DOI:** 10.7759/cureus.5081

**Published:** 2019-07-04

**Authors:** Stephen P Gonzalez, Mitchell S Wachtel, Edwin O Onkendi

**Affiliations:** 1 Surgery, Texas Tech University Health Sciences Center, Lubbock, USA; 2 Pathology, Texas Tech University Health Sciences Center, Lubbock, USA

**Keywords:** gallbladder carcinoma, anastomosing hemangioma, liver, gallbladder, extended cholecystectomy

## Abstract

We report a case of stage T1b gallbladder carcinoma with concurrent hepatic anastomosing hemangioma managed by operative resection. We review the work-up and surgical management of this patient. We also discuss the relevant literature of both gallbladder cancer and hepatic anastomosing hemangioma, a recently described and rare variant of capillary hemangioma.

## Introduction

The history of gallbladder carcinoma has been one of dismal prognosis with a short expected median survival despite advances in chemotherapy and surgical intervention [1]. This has led to more aggressive surgical techniques in the hopes of improving long-term survival in these patients [2-3]. Anastomosing hemangioma is a rare benign vascular tumor. We present a case of early-stage gallbladder carcinoma with incidental anastomosing hemangioma managed by radical operative resection.

## Case presentation

**Figure 1:**
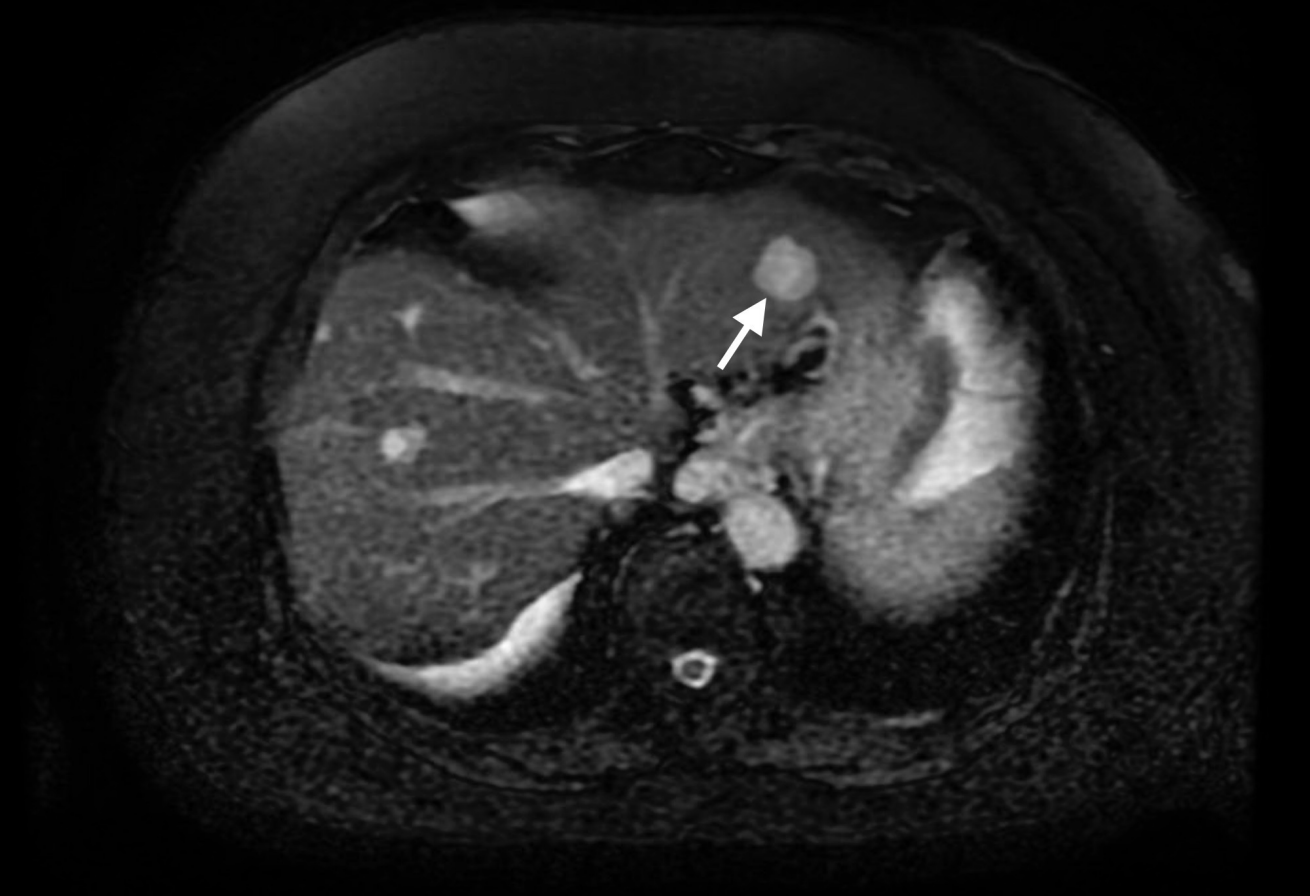
Abdominal computed tomography scan demonstrating 2-cm nodular mass in the left lobe of the liver

An 80-year-old female with a past medical history of hypertension and diabetes mellitus, on oral medication, presented to a community hospital with postprandial right upper quadrant pain and dark stools. She was found to have mild thrombocytopenia. Computed tomography (CT) scan of the abdomen revealed cholelithiasis and an eccentric thickening of the medial wall of the gallbladder concerning for malignancy. There were a few esophageal varices seen; evidence of liver cirrhosis was present. There were no intraabdominal ascites. The patient had no history of alcohol abuse. She was referred to us for further care. Her blood work demonstrated a hemoglobin of 13.9, platelet count of 117,000, total bilirubin 1.4, international normalized ratio (INR) 1.3, and creatinine of 1.1. Her complete hepatitis serology was negative. Upon repeat imaging, her contrast-enhanced CT scan of the abdomen and pelvis revealed a 2 cm liver nodule in the left lateral sector with intense early arterial phase-contrast enhancement, with no early washout or fill-in typical of hepatoma or hemangioma, respectively (Figure 1). Magnetic resonance imaging (MRI) of the abdomen ruled out hemangioma of the left liver lobe, but could not rule out hepatocellular carcinoma or other malignant liver tumors as a possibility. 

**Figure 2:**
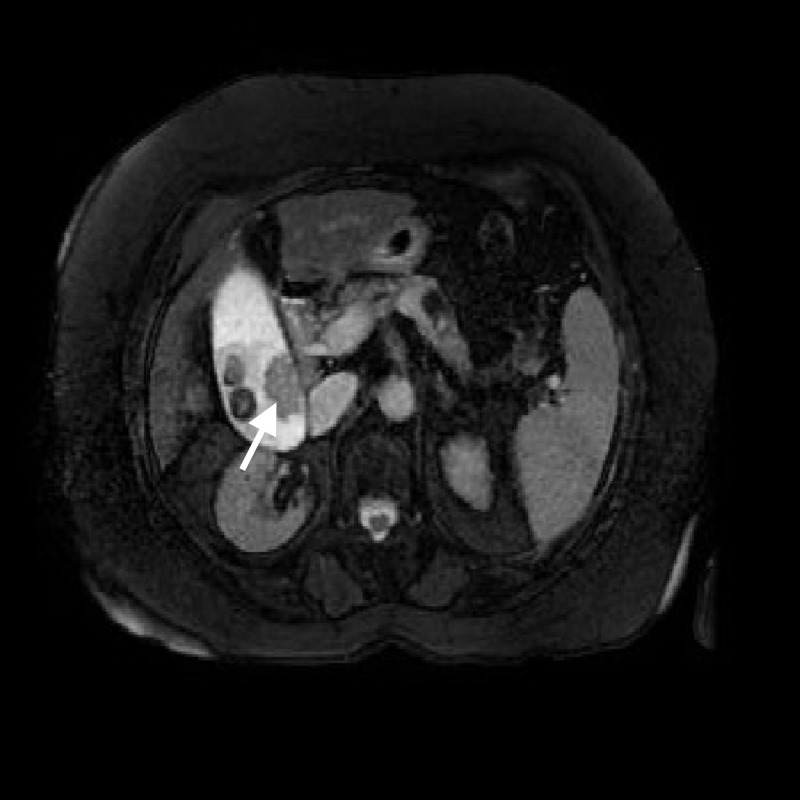
Abdominal computed tomography scan demonstrating a 2.7 x 1.8 cm soft tissue polypoid mass within the gallbladder

There was an enhancing polypoid mass within the gallbladder lumen suggestive of soft tissue on the medial wall of the inferior gallbladder (Figure 2). 

The liver was nodular in contour suggestive of cirrhosis, and paraesophageal varices were present. The gallbladder contained a 2.7 cm x 1.8 cm enhancing mass concerning for malignancy. An upper endoscopy revealed grade two esophageal varices.

**Figure 3:**
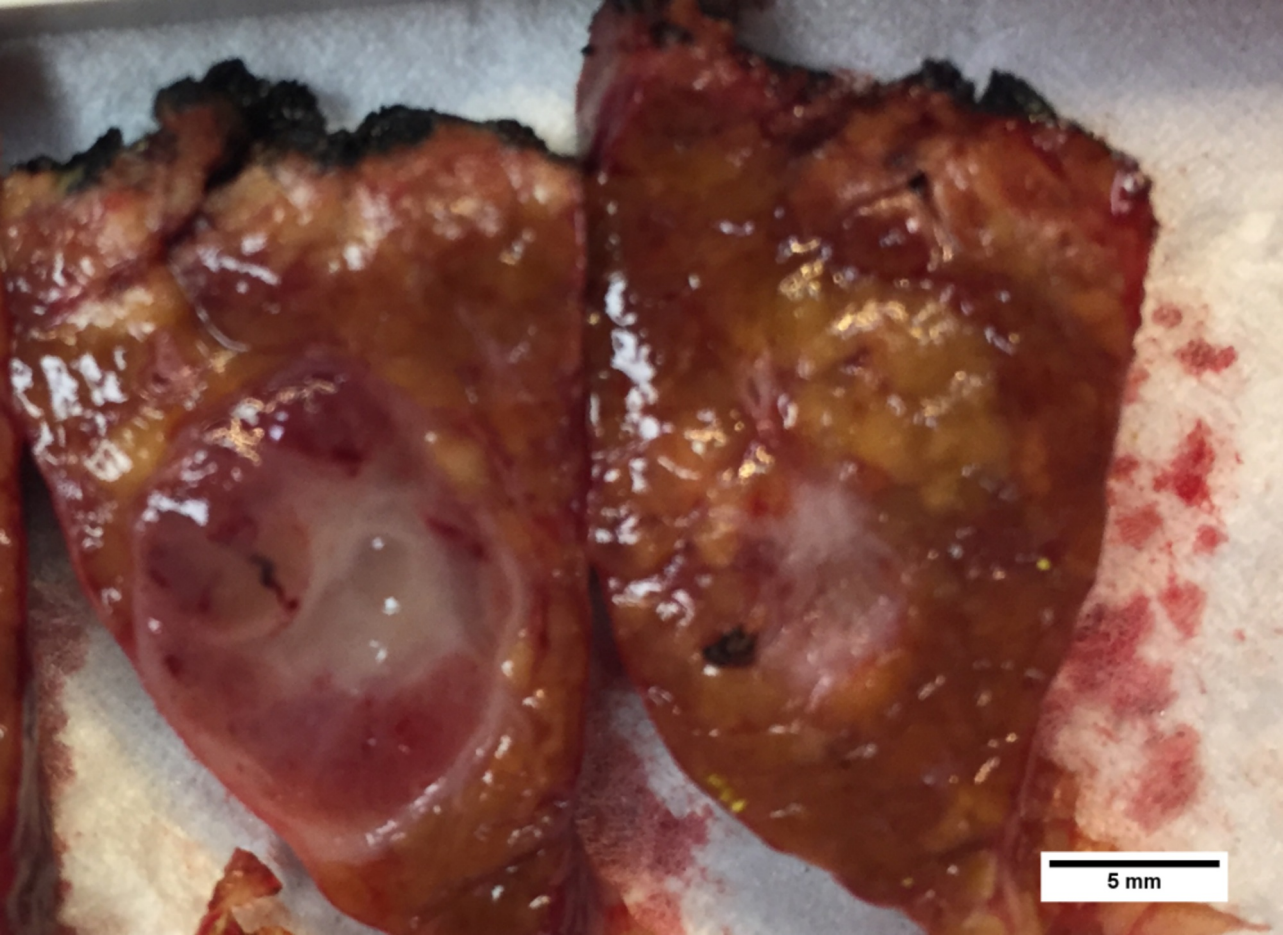
Liver tumor

The patient was determined to have compensated Child-Pugh class A liver cirrhosis with some portal hypertension (small esophageal varices, recanalized umbilical vein, and mild thrombocytopenia) and a model for end‐stage liver disease (MELD) score of twelve. Operative intervention was decided on with the patient. Laparoscopic examination showed no carcinomatosis; however, it showed a nodular liver, and intraabdominal venous varicosities. Laparoscopic liver ultrasonography showed no additional liver masses aside from the known left liver lobe mass. Ultrasonography of the gallbladder showed a hyperechoic intraluminal mass with a hypoechoic rim of interface between it and the medial gallbladder wall. No evidence of invasion into the liver was seen grossly and the gallbladder wall thickness was normal. Therefore, a laparoscopic non-anatomic wedge resection of a portion of left lateral lobe containing the mass and a laparoscopic cholecystectomy were performed. During cholecystectomy, dissection of the gallbladder off the cystic plate was unremarkable and no evidence of transmural extension of tumor into cystic plate was encountered. The left lateral liver resection specimen and gallbladder were submitted for frozen section analysis (Figure 3). This revealed a papillary adenocarcinoma of the gallbladder and a benign vascular tumor of the liver. 

**Figure 4:**
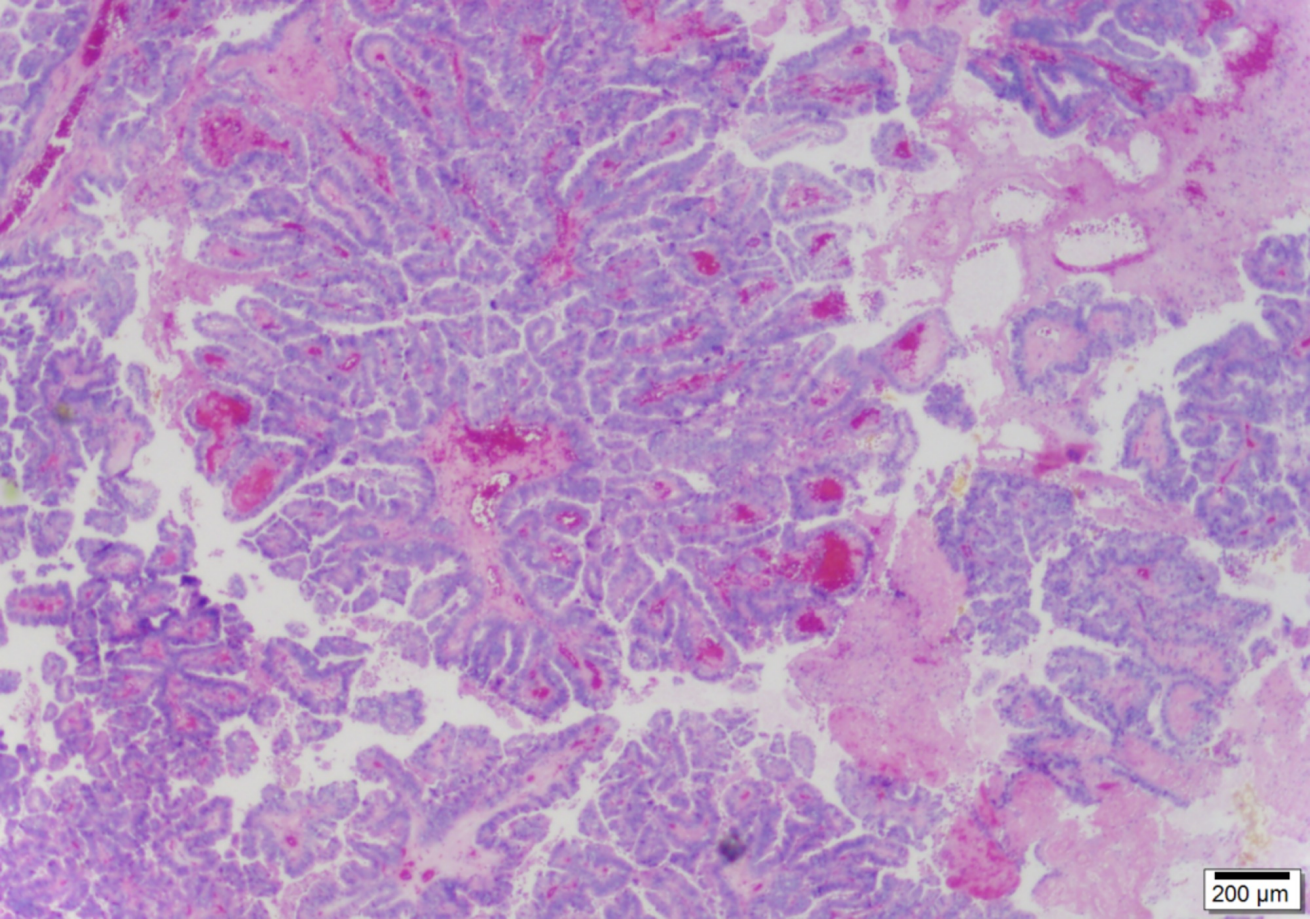
Papillary fronds compose the gallbladder carcinoma (25x)

**Figure 5:**
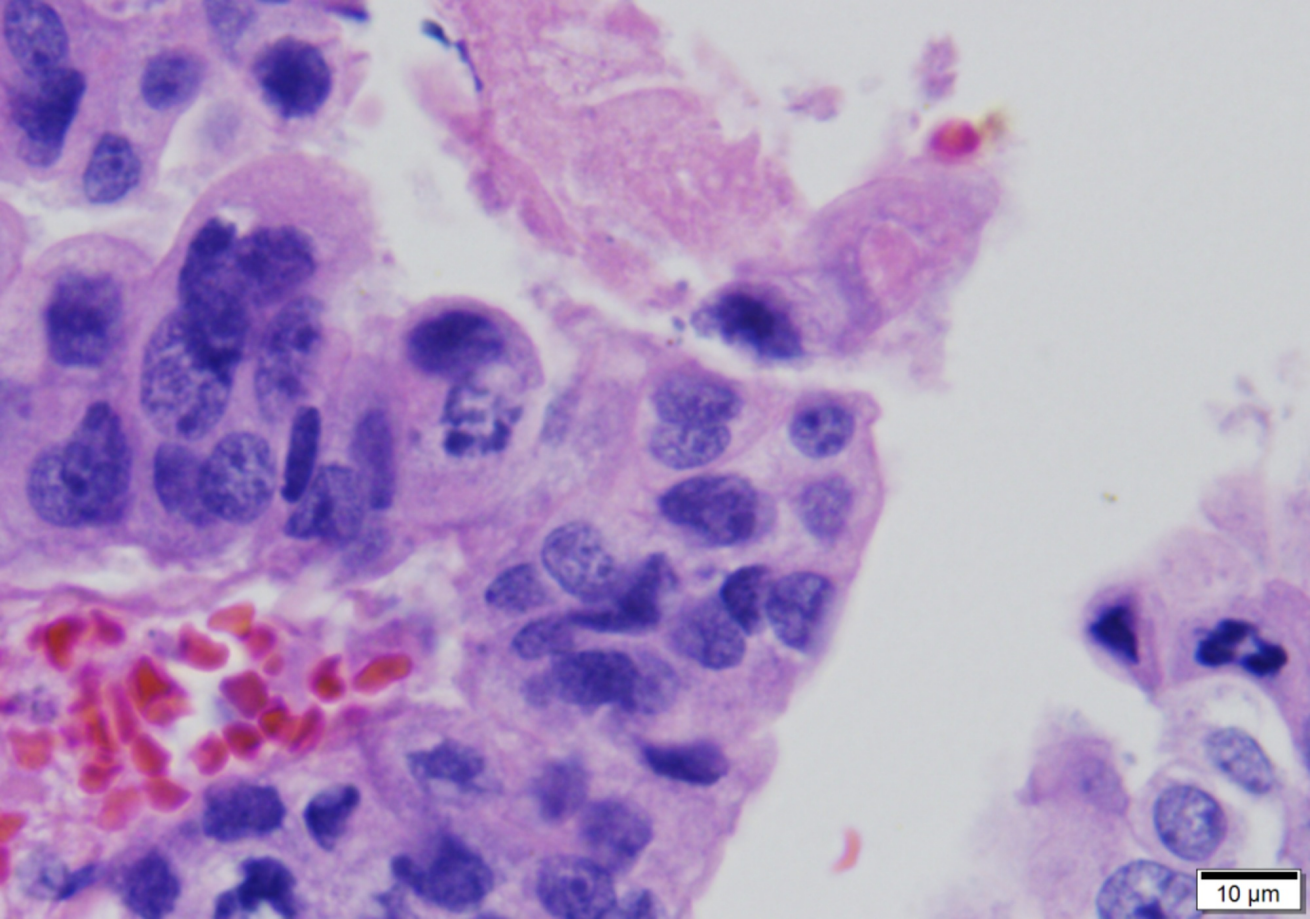
Enlarged, variably sized and shaped lining nuclei showing loss of polarity and aberrant mitoses (630x)

**Figure 6:**
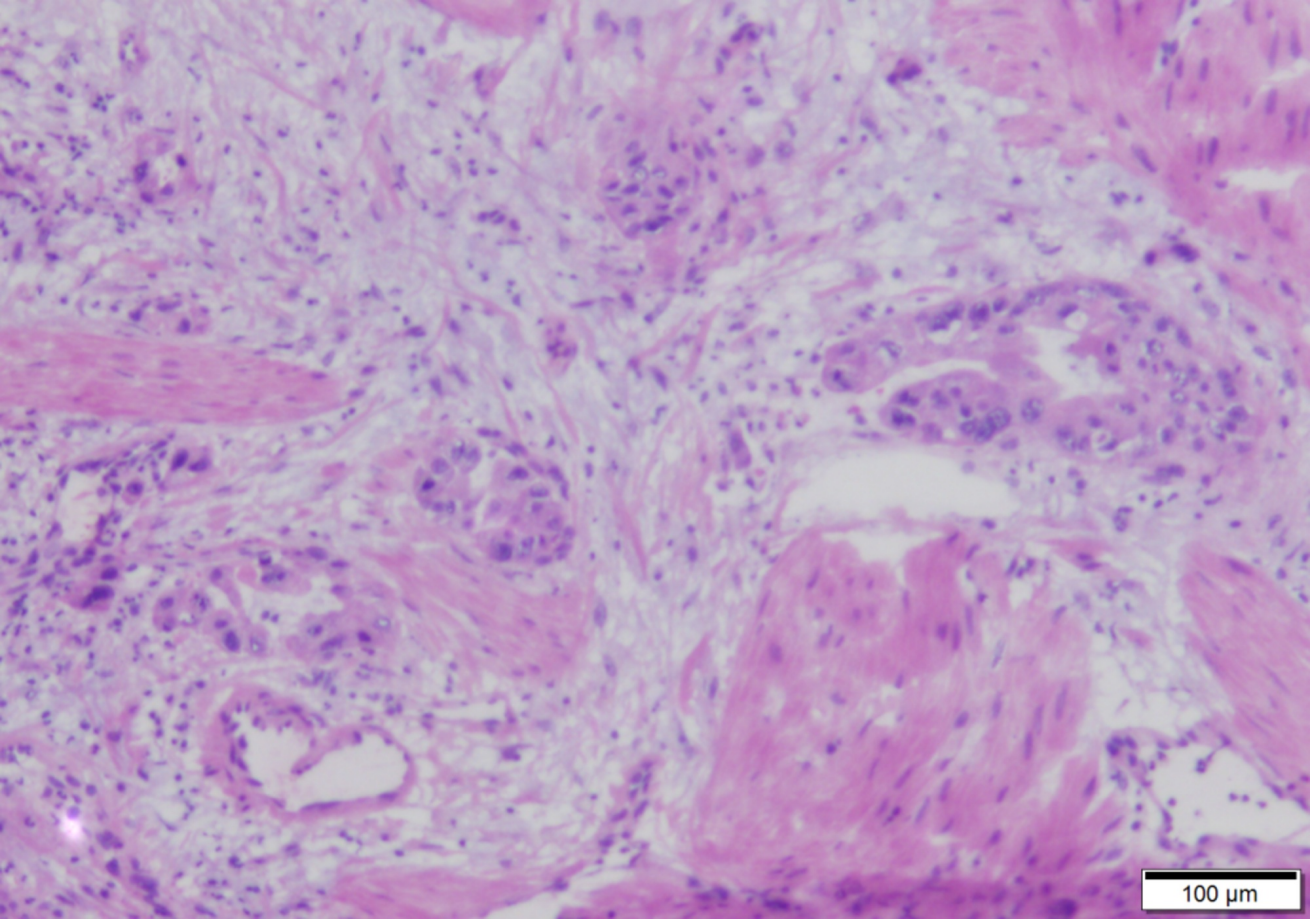
Cancerous glands invade into the muscularis propria of the gallbladder (100x)

As a result of the malignant gallbladder diagnosis with no exact T-staging available on frozen section, a decision was made to perform non-anatomic resection of segments IVb and V en bloc with the cystic plate and hepatoduodenal lymphadenectomy. Therefore, a limited right subcostal incision was made and a non-anatomic resection of portions of segment IVb and V, en bloc with the cystic plate, was performed as well as a hepatoduodenal lymphadenectomy. Cystic duct margin, on frozen section, was negative for malignancy. Final pathology revealed a 2.7 cm T1b well-differentiated grade one papillary carcinoma of the gallbladder; no lymph nodes were found in the specimens (Figures 4-6).

**Figure 7:**
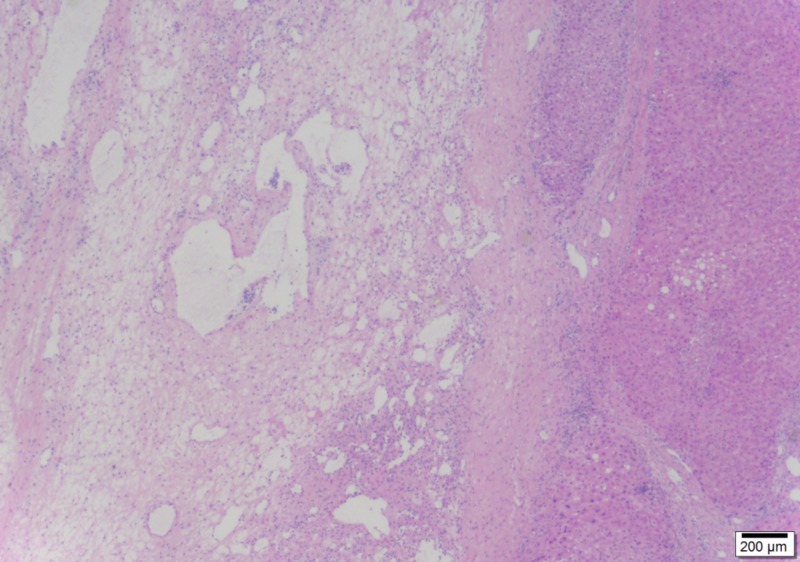
The non-lobulated vascular lesion is sharply demarcated from the liver (25x)

**Figure 8:**
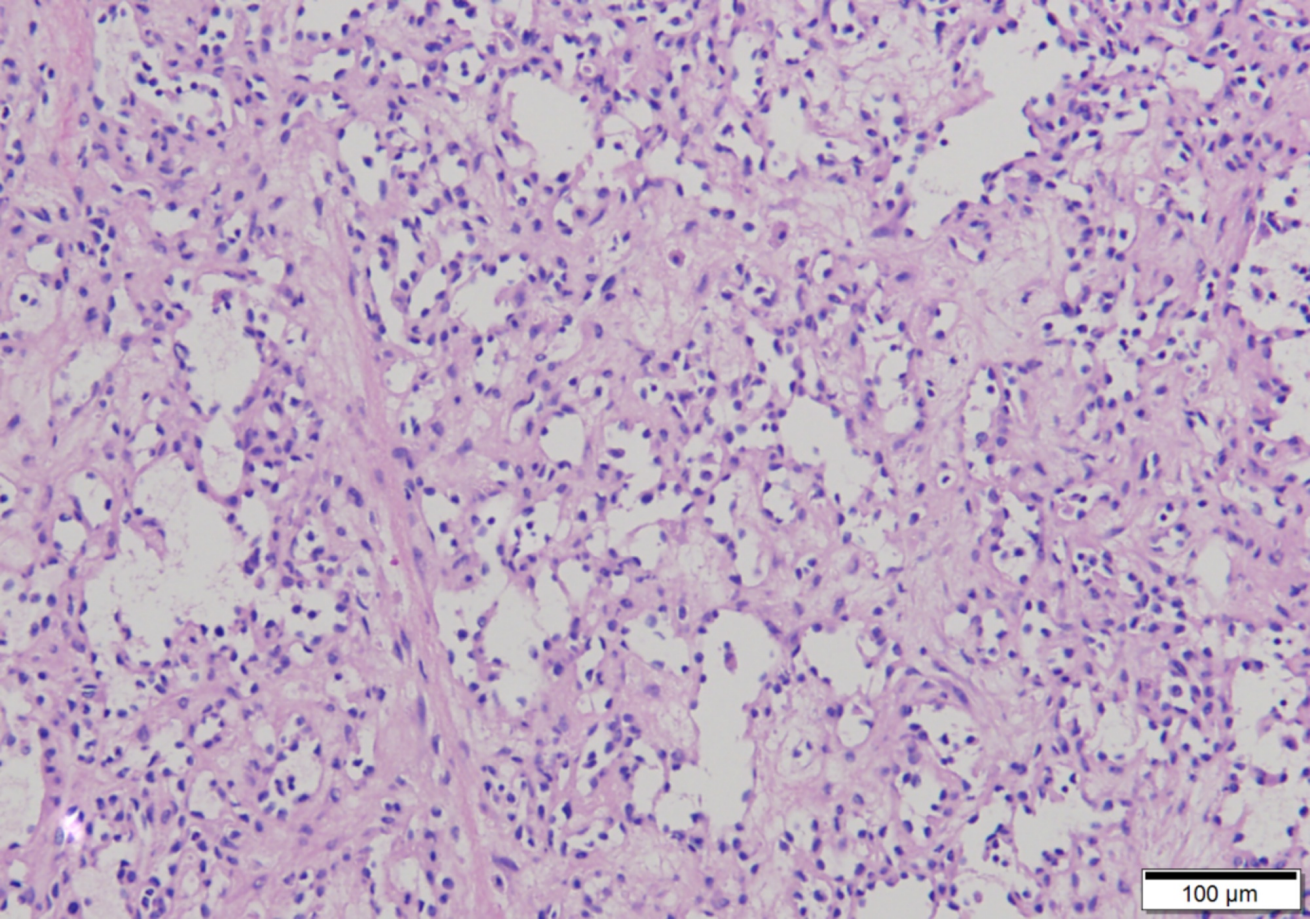
Blood vessels branch, giving rise to the term “anastomosing” (100x)

**Figure 9:**
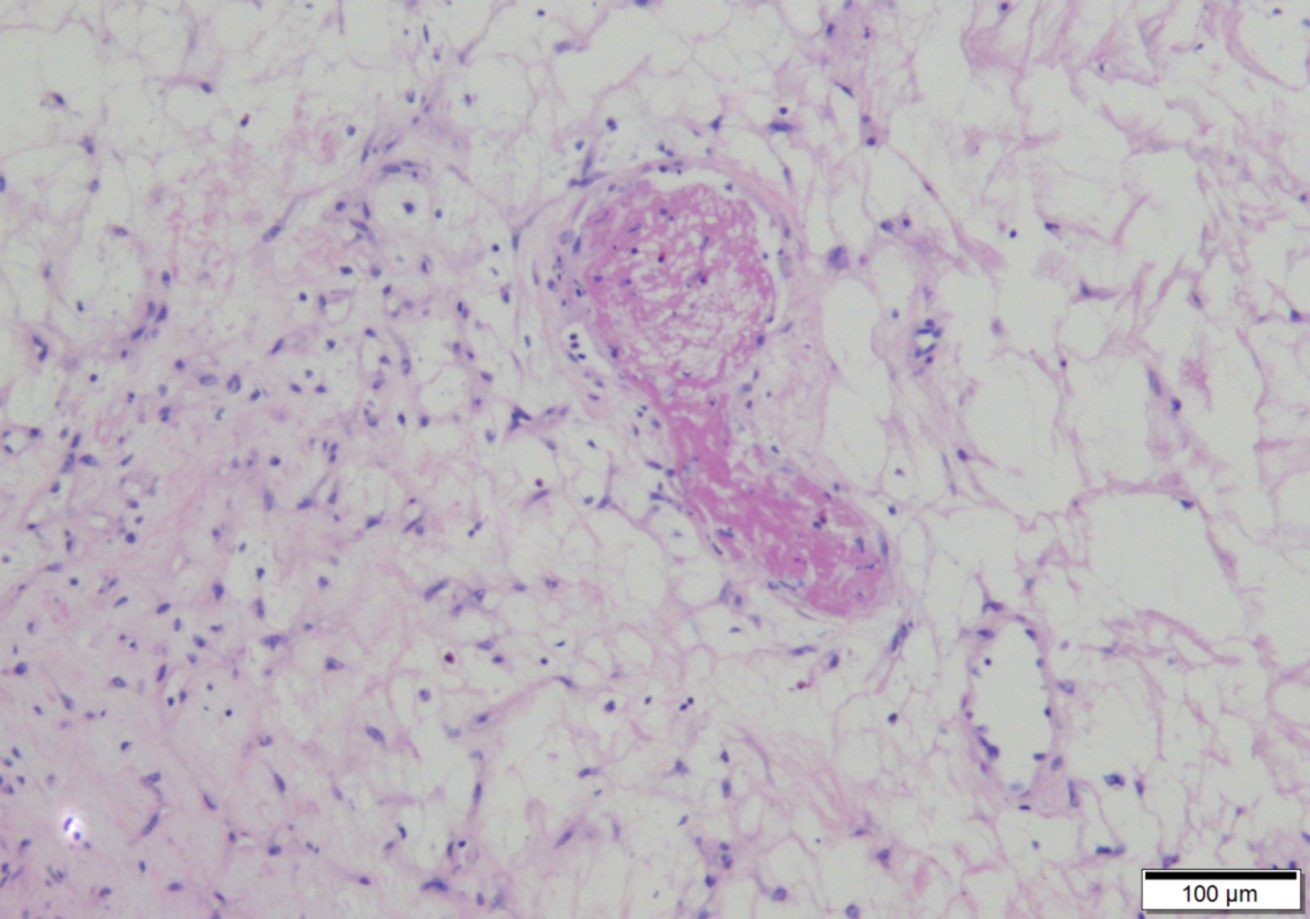
A thrombus lies within the edematous center of the lesion, a characteristic feature (100x)

**Figure 10:**
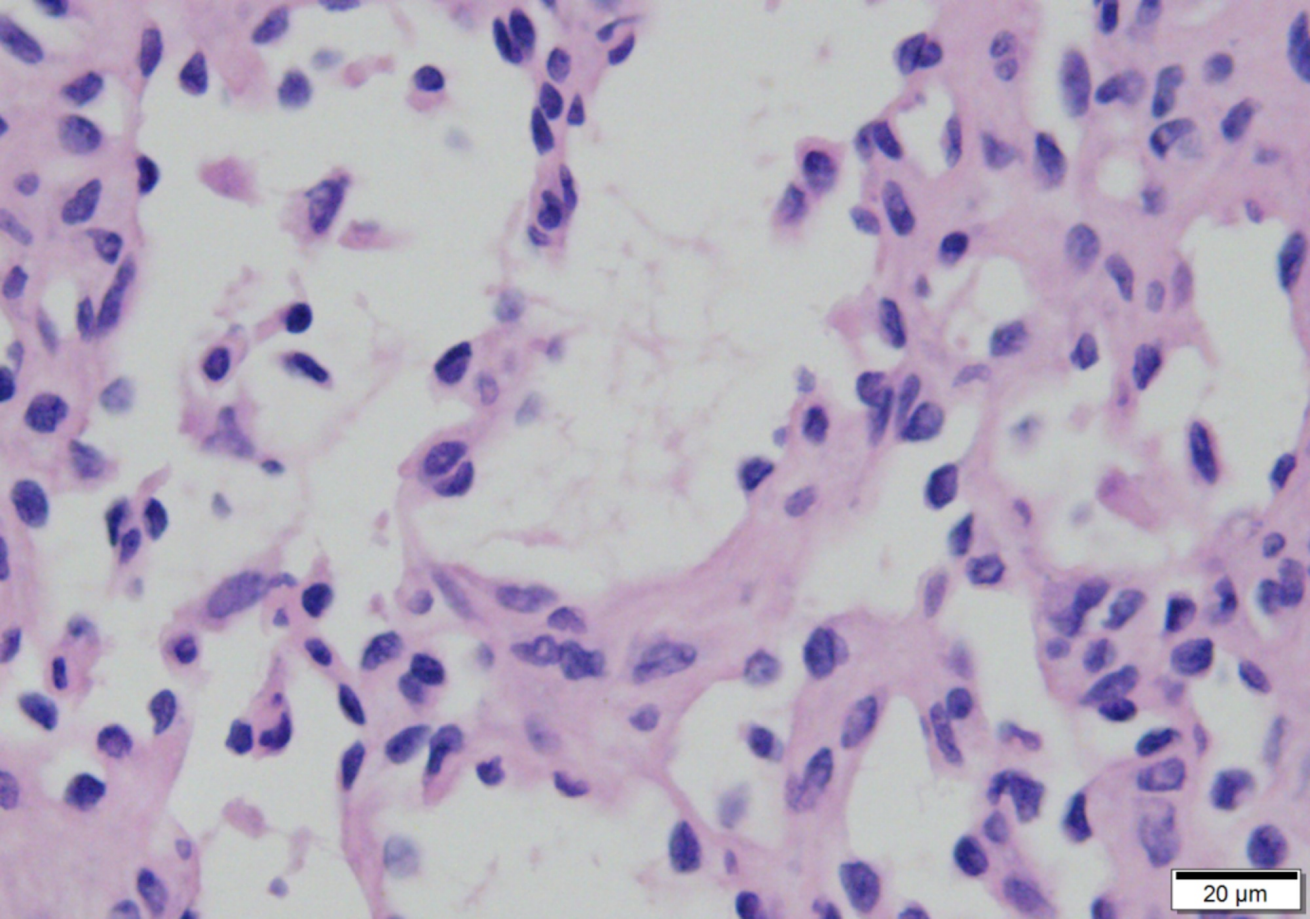
Nuclei showing mild atypia with occasional hobnailing, but mitoses are lacking (400x)

The liver mass was determined to be an anastomosing hemangioma with sharp demarcation from the surrounding liver parenchyma, associated with a branching vascular pattern with mild nuclear atypia and absence of mitoses (Figures 7-10). 

Postoperative recovery was uneventful and the patient was discharged on postoperative day five. She was doing well 18 months postoperatively with no evidence of recurrence.

## Discussion

In this patient, it was not clear whether there was gallbladder malignancy on laparoscopic intraoperative ultrasonography. Although an intraluminal gallbladder hyperechoic mass was visualized, there was no associated gallbladder wall thickening. In addition, on intraoperative ultrasonography, there was a hypoechoic rim/interface all around the hyperechoic mass suggesting lack of origin from or invasion into the gallbladder wall. This guided the decision to perform laparoscopic cholecystectomy first. In addition, the hyperechoic mass in the gallbladder might have been inspissated sludge. Given the fact that there was no clear evidence of invasion on ultrasonography, a laparoscopic cholecystectomy was performed with a plan to analyze the hyperechoic mass by frozen section. Caution was taken to ensure no spillage of gallbladder contents occurred. Once malignancy was confirmed on frozen section, radical resection of portions of segments IVb and V of liver en bloc with the cystic plate and hepatoduodenal lymphadenectomy were performed as above. This was because the exact T-staging of the malignancy was not known at the time of surgery on a frozen section to guide the extent of resection.

Gallbladder carcinoma is a highly aggressive malignancy that carries a poor prognosis. In 1994 a study by Cubertafond et al. [1] estimated a five-year survival rate of 5% and a median life expectancy of three months after diagnosis. However, recent studies suggest that more aggressive surgical management with extended resection may improve long-term survival [2-3]. This more aggressive extended radical approach involves a cholecystectomy, en bloc with wedge resection of the gallbladder fossa and cystic place with a rim of non-neoplastic liver tissue (approximately 2 cm in thickness or more), with/without resection of the suprapancreatic segment of the extrahepatic bile duct and regional lymph node dissection en bloc. This differs from the previously devised Glenn radical cholecystectomy by the extent of regional lymphadenectomy and presence or absence of bile duct resection [4]. Weighed against the dismal median survival, the morbidity and mortality associated with this aggressive therapy are acceptable [5]. It appears that regardless of surgical approach, resection of the tumor with negative margins appears to provide the greatest long-term survival [3-4], even in patients with node-positive disease (when involving one to two nodes) [4].

There is an ongoing debate as to whether simple cholecystectomy alone is adequate, or radical resection with adjacent liver resection and regional lymphadenectomy is superior in managing T1b tumors. Those in favor of radical resection cite evidence of residual cancer being left in the resected liver bed in 46% of T1b tumors treated with prior cholecystectomy [6]. Shirai et al. [4] asserted that this could be due to incomplete excision of the cystic plate disrupting the subserosal plane where tumor cells are left behind. They also recognize that complete excision of the cystic plate facilitates the removal of cystic duct nodes contained within adipose tissue within the triangle of Calot. Bile duct resection is considered optional for tumors pT1b or greater by National Comprehensive Cancer Network guidelines [7]. Given that this patient had negative cystic duct margin, bile duct resection was not required.

Although it remains controversial, the supportive rationale for selecting extended cholecystectomy relies on the evidence that the incidence of lymph node metastasis and recurrence rate are both significantly higher in T1b gallbladder cancer than T1a [8]. There is also some evidence that radical resection demonstrates improved long-term survival in patients with T1b tumors when compared to simple cholecystectomy alone [9]. However, Jang et al. [10] argue that there are problems with the studies included in the meta-analysis, and found similar survival and recurrence rates in patients with T1b and T1a. They also supported laparoscopic resection. Less blood loss, shorter hospital stays, and better cosmetic outcomes are considered to be advantages of the laparoscopic approach.

Without definitive evidence suggesting one approach is superior, it is up to the surgeon’s judgment and the results of frozen section analysis at the time of the procedure to guide decision-making in the management of early-stage gallbladder carcinoma. However, like in this patient, the exact T-staging of the tumor is often not available at the time of frozen section to guide the extent of resection. So it is wiser to err on the side of extended resection than simple cholecystectomy when an intraoperative diagnosis of gallbladder cancer is made. If a postoperative diagnosis of gallbladder cancer is made, then T-staging can guide decision-making on the need for reoperation for extended resection.

The left hepatic vascular tumor was confirmed to be an anastomosing hemangioma after analysis by the second senior co-author (MSW) with the additional expert review by Dr. Andrew Folpe of the Mayo Clinic. Anastomosing hemangioma is a rare vascular tumor first described by Montgomery and Epstein in 2009 [11], with more than 50 cases reported in the kidney and near as many in other organs since [12]. Available literature all endorses the benign nature of the neoplasm [12-17], with one long-term case study of a disease-free interval of 13 years following nephrectomy [16]. The primary differential consideration for diagnosing anastomosing hemangioma is differentiating angiosarcoma, a highly aggressive malignancy requiring vastly different management. Features of anastomosing hemangioma are characteristic. Macroscopically, it is mahogany-colored with a spongy consistency and well-defined margins [12-15,17]. Microscopically, it consists of anastomosing sinusoidal-like spaces lined by a single file of CD31/CD34+ endothelial cells, some with hobnail morphology, supported by pericytes, frequently associated with intravascular thrombi and features of extramedullary hematopoiesis [12-17]. Anastomosing hemangioma appears sharply demarcated with mild cytologic atypia and lacks mitotic figures in contrast to angiosarcoma which displays prominent cytologic alterations and a diffuse infiltrating border [12-15,17].

This patient’s early stage gallbladder carcinoma completely resected with negative margins improves her chance of five-year survivability [2]. The National Comprehensive Cancer Network guidelines suggest imaging be “considered” every six months for two years as clinically indicated [7]. However, there are no evidence-based guidelines for appropriate follow-up after treatment of gallbladder cancer and no data supports aggressive posttreatment surveillance.

## Conclusions

The surgical approach for stage T1b gallbladder carcinomas is controversial, with no definitive evidence demonstrating the superiority of extended radical cholecystectomy over routine laparoscopic cholecystectomy. Anastomosis hemangioma, a subtype of cavenous hemangioma, can be confused with angiosarcoma. However, anastomosing hemangioma has well-defined margins and appears sharply demarcated with mild cytologic atypia and lack mitotic figures, in contrast to angiosarcoma which displays prominent cytologic alterations and a diffuse infiltrating border.
